# Establishment of the 3M syndrome animal model in CCDC8 knockout mice

**DOI:** 10.1186/s43556-023-00136-0

**Published:** 2023-08-14

**Authors:** Lei Zhang, Doudou Ren, Xiaoyan Hu, Jinhuan Sun, Chunxia Qi, Yanfeng Wang, Lingling Lu, Min Wei

**Affiliations:** School of Medicine, Nankai University, Tianjin, P.R. China

**Keywords:** 3M syndrome, CCDC8, Homozygote, Heterozygote, Animal model

Dear Editor,

3M syndrome is named from the initial letters of the scientists who first described this disorder, Miller, McKusick, and Malvaux in 1975 [1]. 3M syndrome, or called 3M dwarfism, is an autosomal recessive disorder characterized by severe pre- and postnatal growth retardation (adult height ~ 115-150 cm) [2, 3]. Additional features of 3M syndrome patients include slender long bones, fleshy tipped nose, deformed sternum, square shoulders, winged scapulae, hyperlordosis, prominent heels, loose joints and other skeletal abnormalities [2, 3]. 3M patients present normal intelligence, and female 3M patients can become pregnant. Globally, more than 100 cases have been reported, and in recent years, more cases are diagnosed and reported [2, 3].

Exome sequencing revealed that 3M syndrome was related to the mutations of E3 ligase *Cul7* (Cullin 7), or cytoskeleton protein *Obsl1* (obscurin-like protein 1), or *CCDC8* (coiled-coil domain containing protein 8) in a mutually exclusive manner [3]. Human 3M syndrome is therefore subcategorized into 3 subtypes 3M1, 3M2, and 3M3, respectively related to the mutations of *Cul7*, *Obsl1*, and *CCDC8*. Around 67% 3M patients have *Cul7* mutations, 28% have *Obsl1* mutations, and less than 5% have *CCDC8* mutations [2, 3]. Among them, CCDC8-mutated 3M syndrome patients are mild with higher height than Obsl1- or Cul7- mutated patients. A line of evidence indicates that CCDC8, Obsl1, and Cul7 are in a same pathway [2–5].

Until now, there is no successful animal model for 3M syndrome, such as mouse, rabbit, or monkey model. Cul7 or Obsl1 knockout mice were unsuccessful, because Obsl1 knockout was embryonically lethal, and Cul7 knockout mice died immediately after birth due to respiratory distress [6, 7]. It was reported that an inherited disorder in Australian Poll Merino/Merino sheep, called brachygnathia, cardiomegaly and renal hypoplasia syndrome (BCRHS), was related with the variant of Obsl1 [8]. BCRHS-affected lambs are stillborn with various defects such as short stature, a short and broad cranium, similar to 3M syndrome. The BCRHS sheep is a disease model, but not a live 3M syndrome model [8]. Wang P, et al. tried to use homologous recombination to knockout CCDC8 in mice. It was still unsuccessful, and they did not obtain viable CCDC8-knockout mice [9].

The aim of this study is to establish the 3M syndrome animal model, and explore the underline mechanism. In this study, we successfully obtained CCDC8-knockout mice using CRISPR-Cas9 (**C**lustered **R**egularly **I**nterspaced **S**hort **P**alindromic **R**epeats, **C**RISPR **as**sociated nuclease 9) genome editing technology.

Because mouse CCDC8 gene locus is on the chromosome 7 and includes only one exon (https://www.ncbi.nlm.nih.gov/), two sgRNAs were designed in the flank region of CCDC8 open reading frame to get CCDC8-knockout mice (supplementary Fig. 1a, b). The CCDC8-knockout sperms were microinjected into mouse eggs to get F0 generation of mice. The positive founder mice were collected and the information was listed in supplementary Table 1. Through crossbreeding with wild type C57BL/6 J mice, we obtained four knockout (KO) positive heterozygous CCDC8^+/-^ mice, in which 2996 bp or 3000 bp of CCDC8 open reading frame was completely deleted (supplementary Table 2). This genotype was confirmed by PCR and Sanger sequencing (supplementary Fig. 1c-g).

To get homozygous CCDC8-knockout mice, we continued to crossbreed heterozygous CCDC8^±^ mice. Finally, we obtained 4 homozygous CCDC8^−/−^ mice (supplementary Table 3). The genotype was also confirmed by PCR and sequencing (Fig. 1a, b, c). Whatever we tried to mate homozygous CCDC8^−/−^ female mice with homozygous CCDC8^−/−^ male mice or heterozygous CCDC8^+/-^ mice or vise versa, we only obtained 4 homozygous CCDC8^−/−^ mice, two female and two male mice until January 2020, in total 410 mice (supplementary Table 4).

**Fig. 1:**
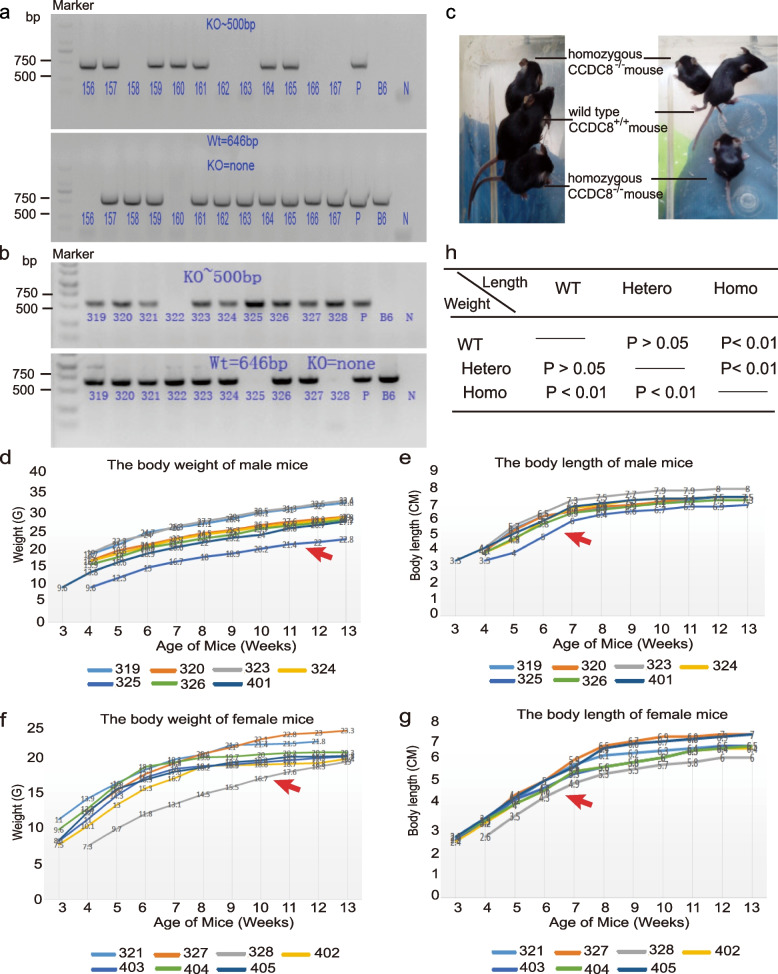
CCDC8 knockout mice. **a** **b** PCR products of pair 1 primers (upper panel) and pair 3 primers (lower panel) were analyzed in agarose gel electrophoresis. The numbers of mouse samples were indicated. Marker, DNA marker; P, positive control; B6, wild type control; N, negative control. **c** Pictures of CCDC8 knockout mice CCDC8 ^−/−^ and wild type CCDC8 ^+/+^ mouse at the same age of 3 weeks. **d** **e** **f** **g** The growth curve of male and female mice. The body weight (**d**) and length (**e**) of male mice. Male CCDC8 knockout mice CCDC8^−/−^ (325, red arrow); male CCDC8^+/-^ (319, 323, 324, 326); male wild type CCDC8^+/+^ (320, 401). The body weight (**f**) and length (**g**) of female mice. Female CCDC8 knockout mice CCDC8^−/−^ (328, red arrow); female CCDC8^+/-^ (327, 402, 403, 404); female wild type CCDC8^+/+^ (321, 405). **h** The Wilcoxon-Rank-Sum test results of weight and length in CCDC8^−/−^ (*n* = 2), CCDC8^+/-^ (*n* = 8), and CCDC8^+/+^ mice (*n* = 8). WT, wild type CCDC8^+/+^ mice; Hetero, heterozugous CCDC8^±^ mice; Homo, homozygous CCDC8^−/−^ mice

Mouse body length and weight from week 3 to week 13 were listed in Fig. 1d, e, f, g. The body length and weight of the male and female homozygous CCDC8^−/−^ mice were significantly lower than that of heterozygous CCDC8^+/-^ or wild-type CCDC8^+/+^ mice, but there was no difference between CCDC8^+/-^ and CCDC8^+/+^ mice (Fig. 1d-h). CCDC8^−/−^ mice still grew, but the weight and length were lower than CCDC8^+/-^ and CCDC8^+/+^ mice (Fig. 1d-h). These characteristics resemble 3M patients in humans. Through our naked eye observation, no apparent difference was found in food eating, behavior, mental state, and health status between CCDC8^−/−^ mice and CCDC8^+/-^, CCDC8^+/+^ mice (Supplemental movie). In addition, CCDC8^−/−^ female mice can become pregnant and CCDC8^−/−^ male mice are fertile. When they breed with wild type mice respectively, they can produce mice as normal. However, CCDC8^−/−^ male and CCDC8^−/−^ female mice can not produce viable births: the CCDC8^−/−^ female mice had spontaneous abortion before birth, or the baby mice died soon after birth. The heterozygous CCDC8^+/-^ female mice were able to conceive and give birth, and four CCDC8^−/−^ mice were born by CCDC8^+/-^ mother and CCDC8^+/- ^father. Similarly, when CCDC8^−/−^ female mice and CCDC8^+/-^ male mice were mated, and CCDC8^−/−^ female mice were pregnant, but they still either had spontaneous abortion, or the baby mice died soon after birth. Until now, only one mouse born by a CCDC8^−/−^ female mouse and a ﻿﻿CCDC8^+/-^﻿ male mouse, and the genotype of this mouse was heterozygous CCDC8^+/-^.

Although CCDC8^+/-^ female mice and CCDC8^+/-^ male mice can give birth, the number per litter was 3–4 mice, maximum 8 mice, compared to 6–15 mice per litter in CCDC8^+/+^ wild type mice.

The life expectancy of all homozygous CCDC8^−/−^ mice is more than 1 year with no difference from wild type CCDC8^+/+^ mice or heterozygous CCDC8^+/-^ mice.

In this study, CCDC8-knockout mice were successfully produced using CRISPR-Cas9 genome editing technology. Genotyping was confirmed by PCR and sequencing. CCDC8^−/−^ mice present the lower body weight and length than CCDC8^+/-^ and CCDC8^+/+^ mice, and still grow. But behavior, mental state of CCDC8^−/−^ mice does not change apparently. CCDC8^−/−^ female mice can become pregnant and give birth, although they have a high spontaneous abortion when mating with CCDC8^−/−^ male or CCDC8^+/-^ heterozygous male mice. The life span of CCDC8^−/−^ mice is more than 1 year, like the CCDC8^+/-^ and CCDC8^+/+^ mice. All of these characteristics resemble human 3M syndrome [1–3]. Therefore, we believe a viable 3M syndrome mouse model was successfully established.

In total 410 mice in this study, we only produced four live CCDC8^−/−^ mice, and the success rate is less than 1%. The lower rate could be the fact reflection of lower incidence rate of more than 100 reported human cases in total 7.5 billion people globally (~ 0.0013/100,000). These data indicate that CCDC8 is an important gene for human and mouse growth. Knockout of CCDC8 is lethal to some extent, although fewer cases can bypass its malfunction and give birth to humans or mice with no lethal or life-threatening problems. The 3M patient with CCDC8 mutation was reported with c.612insG, resulting in a premature N terminal 38KD CCDC8 protein [3]. This mutated CCDC8 loses most of C terminal protein in 90KD full length CCDC8 protein, which is comparable with CCDC8-knockout mice. CCDC8-mutated 3M syndrome patients are mild with higher height than Obsl1- or Cul7- mutated patients. This phenomenon suggests that CCDC8-konckout is less lethal than Obsl1- or Cul7-konckout.

The underline mechanism of 3M syndrome is still not clear. More evidence supports that CCDC8-Obsl1-Cul7 complex play an important role in 3M syndrome [3]. CCDC8 is a membrane-associated protein, and N-terminal of CCDC8 harbors plasma membrane localization signal [4]. 3M patients are usually resistant to growth hormone (GH) and/or Insulin-like Growth Factor 1 (IGF-1) therapy [10]. However, Insulin-like Growth Factor 2 (IGF-2) is down-regulated in 3M patients [10]. Therefore, IGF-2 related plasma membrane signaling pathway is more likely to be the mechanism of 3M syndrome. The mechanism of CCDC8-Obsl1-Cul7-IGF2 pathway on human and mouse growth is worth to be investigated deeply in the future.

In conclusion, this study established a 3M syndrome mouse model, CCDC8-knockout mice. This 3M syndrome animal model can be a quite important tool to further investigate the mechanism of 3M syndrome, and the therapy of 3M syndrome in the future.

### Supplementary Information

**Additional file 1: Supplementary Figure 1.** Strategy for CCDC8 knockout mice. (a) Schematic illustration of CCDC8 knockout by CRISPR-Cas9 genome editing technology. ATG, initial codon of translation; TAA, stop codon of translation; UTR, untranslated regions. (b) sgRNA (single guide RNA) sequences for CCDC8 knock out. PAM, protospacer adjacent motifs. (c)(e) PCR products of pair 1 primers were analyzed in agarose gel electrophoresis. The numbers of mouse samples were indicated. M, DNA marker; WT, wild type; NC, negative control. (d)(f) PCR products of pair 2 primers were analyzed in agarose gel electrophoresis. The numbers of mouse samples, same as (c),(e) were indicated. (g) Sequencing results of the mouse sample 66, 68 with 2996 bp deletion, and sample 69, 71 with 3000 bp deletion. **Table 1**. Information of eight positive KO founder mice. **Table 2.** F1 generation mice. **Table 3.** Some F2, F3 generation of CCDC8-knockout mice. **Table 4.** Information of mouse breeding. **Table 5.** Primers for PCR.

**Additional file 2.**

## Data Availability

All data generated or analysed during this study are included in this published article and its supplementary information files.

## References

[1] Miller JD, McKusick VA, Malvaux P, Temtamy S, Salinas C (1975). The 3-M syndrome: a heritable low birthweight dwarfism. Birth Defects Orig Artic Ser.

[2] Hanson D, Murray PG, Coulson T, Sud A, Omokanye A, Stratta E (2012). Mutations in CUL7, OBSL1 and CCDC8 in 3-M syndrome lead to disordered growth factor signalling. J Mol Endocrinol.

[3] Hanson D, Murray PG, O'Sullivan J, Urquhart J, Daly S, Bhaskar SS (2011). Exome sequencing identifies CCDC8 mutations in 3-M syndrome, suggesting that CCDC8 contributes in a pathway with CUL7 and OBSL1 to control human growth. Am J Hum Genet.

[4] Jiang X, Jia X, Sun J, Qi C, Lu L, Wang Y (2020). Overexpressed coiled-coil domain containing protein 8 (CCDC8) mediates newly synthesized HIV-1 Gag lysosomal degradation. Sci Rep.

[5] Wei M, Zhao X, Liu M, Huang Z, Xiao Y, Niu M (2015). Inhibition of HIV-1 assembly by coiled-coil domain containing protein 8 in human cells. Sci Rep.

[6] Arai T, Kasper JS, Skaar JR, Ali SH, Takahashi C, DeCaprio JA (2003). Targeted disruption of p185/Cul7 gene results in abnormal vascular morphogenesis. Proc Natl Acad Sci USA.

[7] Blondelle J, Marrocco V, Clark M, Desmond P, Myers S, Nguyen J (2019). Murine obscurin and Obsl1 have functionally redundant roles in sarcolemmal integrity, sarcoplasmic reticulum organization, and muscle metabolism. Commun Biol.

[8] Woolley SA, Hayes SE, Shariflou MR, Nicholas FW, Willet CE, O'Rourke BA (2020). Molecular basis of a new ovine model for human 3M syndrome-2. BMC Genet.

[9] Wang P, Yan F, Li Z, Yu Y, Parnell SE, Xiong Y (2019). Impaired plasma membrane localization of ubiquitin ligase complex underlies 3-M syndrome development. J Clin Investig.

[10] Murray PG, Hanson D, Coulson T, Stevens A, Whatmore A, Poole RL (2013). 3-M syndrome: a growth disorder associated with IGF2 silencing. Endocr Connect.

