# Supplementary material for: Establishment of the 3M syndrome animal model in CCDC8 knockout mice
Source: Mol Biomed. 2023 Aug 14;4:24. doi: 10.1186/s43556-023-00136-0 (PMC10423707; doi:10.1186/s43556-023-00136-0)
Supplement: Supplementary file 1 — Additional file 1: Supplementary Figure 1. Strategy for CCDC8 knockout mice. (a) Schematic illustration of CCDC8 knockout by CRISPR-Cas9 genome editing technology. ATG, initial codon of translation; TAA, stop codon of translation; UTR, untranslated regions. (b) sgRNA (single guide RNA) sequences for CCDC8 knock out. PAM, protospacer adjacent motifs. (c)(e) PCR products of pair 1 primers were analyzed in agarose gel electrophoresis. The numbers of mouse samples were indicated. M, DNA marker; WT, wild type; NC, negative control. (d)(f) PCR products of pair 2 primers were analyzed in agarose gel electrophoresis. The numbers of mouse samples, same as (c),(e) were indicated. (g) Sequencing results of the mouse sample 66, 68 with 2996 bp deletion, and sample 69, 71 with 3000 bp deletion. Table 1. Information of eight positive KO founder mice. Table 2. F1 generation mice. Table 3. Some F2, F3 generation of CCDC8-knockout mice. Table 4. Information of mouse breeding. Table 5. Primers for PCR. [file 43556_2023_136_MOESM1_ESM.docx]

**Establishment of the 3M syndrome animal model in CCDC8 knockout mice**

Lei Zhang^1^, Doudou Ren^1^, Xiaoyan Hu^1^, Jinhuan Sun^1^, Chunxia Qi^1^, Yanfeng Wang^1^, Lingling Lu^1^, Min Wei^1,^

1. School of Medicine, Nankai University, Tianjin, P.R. China

Correspondence author: Lingling Lu, lulingling@nankai.edu.cn

Min Wei, weimin@nankai.edu.cn

**Materials and Methods**

*CCDC8-knockout mice*

Using CRISPR-Cas9 genome editing technology, we tried to get CCDC8-knockout C57BL/6J mice (Model Animal Research Center of Nanjing University, Nanjing, China) (Mouse CCDC8 gene: Gene bank number NC_000073.6, genome assembly GRCm38.p6). Mice are raised in SPF (Specific Pathogen Free) feeding room. The temperature in the feeding room is 22-28 °C and the relative humidity is 40-60%. Alternating time of day and night is 12/12 hours. Indoor noise is less than 60dB. Indoor ammonia concentration can not exceed 20ppm, and the ventilation times must reach 10-20 times/hour. The mouse food is sterilized by ^60^Co γ ray. Drinking water must be autoclaved. Keep enough fresh food and water in the feeding box. The padding of feeding box is mixed autoclaved sawdust.

*DNA extraction and PCR amplification*

Mouse DNA was extracted by Mouse Tail Genomic DNA Kit according to the manufacturer’s standard protocol (CW2094S, CWBio, China). To identify the genotype, PCR (Polymerase Chain Reaction) was carried out. The PCR primer sequences are listed in supplementary Table 5. Pair 1 primer binding sites were designed in the flank region of CCDC8 open reading frame, located at the outside of CRISPR/Cas9 DNA breaking sites (supplementary Fig. 1a). The size of pair 1 PCR products for wild type CCDC8 is 3512bp, but it is around 500bp for knockout (KO) positive CCDC8. PCR condition was set to 94°C for 30 seconds, 58°C for 30 seconds, and extend at 72°C for 30 seconds for 30 cycles. Thus, under these PCR conditions, wild type 3512bp PCR products did not appear on the agarose gel. The pair 1 primers were used to exclude the wild type CCDC8. Pair 2 and 3 primers were designed in the internal region of the CCDC8 open reading frame. The PCR products of pair 2 and 3 primers for wild type CCDC8 are 819bp and 646bp respectively, but KO positive CCDC8 shows no bands (supplementary Table 5). Therefore, our strategy was to use pair 1 primers to exclude wild type CCDC8, and pair 2 or 3 primers to identify homozygous or heterozygous CCDC8.

*Measurement of mouse weight and body length*

From 3 to 13 weeks after their birth, mouse weight and body length were recorded. Mouse weight was measured by an electric scale. The mouse body length is the distance between the middle of two ears and the base of mouse-tail. Mouse behavior and fertility were also observed by naked eyes, recorded and compared by the same technician.

*Statistical analysis*

Non-parametric test (Wilcoxon-Rank-Sum test) was applied for statistical analysis.

**
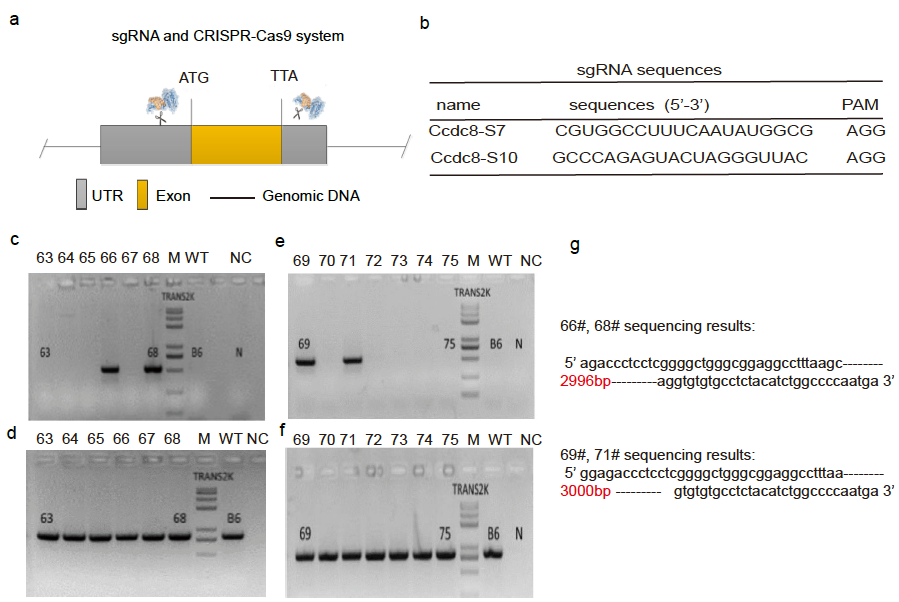
**

**Supplementary Figure 1. Strategy for CCDC8 knockout mice.** (**a**) Schematic illustration of CCDC8 knockout by CRISPR-Cas9 genome editing technology. ATG, initial codon of translation; TAA, stop codon of translation; UTR, untranslated regions. (**b**) sgRNA (single guide RNA) sequences for CCDC8 knock out. PAM, protospacer adjacent motifs. (**c**)(**e**) PCR products of pair 1 primers were analyzed in agarose gel electrophoresis. The numbers of mouse samples were indicated. M, DNA marker; WT, wild type; NC, negative control. (**d**)(**f**) PCR products of pair 2 primers were analyzed in agarose gel electrophoresis. The numbers of mouse samples, same as C, E, were indicated. (**g**) Sequencing results of the mouse sample 66, 68 with 2996 bp deletion, and sample 69, 71 with 3000 bp deletion.

**Table 1. Information of eight positive KO founder mice**

| ID | Gender | Color | Gty | DOB | Gen | |
| --- | --- | --- | --- | --- | --- | --- |
| 10 | ♀ | B | + | 2016/12/13 | F0 |  |
| 21 | ♀ | B | + | 2016/12/13 | F0 |  |
| 28 | ♂ | B | + | 2016/12/13 | F0 |  |
| 31 | ♀ | B | + | 2016/12/13 | F0 |  |
| 32 | ♀ | B | + | 2016/12/13 | F0 |  |
| 42 | ♀ | B | + | 2016/12/16 | F0 |  |
| 55 | ♂ | B | + | 2016/12/16 | F0 |  |
| 60 | ♀ | B | + | 2016/12/16 | F0 |  |

ID, Identification number; ♀, female; ♂, male; B, Black; Gty, Genotype; DOB, Date of birth (year/month/date); Gen, Generation.

**Table 2. F1 generation mice**

| ID | Gender | DOB | Color | Gty | F/M | Gen | |
| --- | --- | --- | --- | --- | --- | --- | --- |
| 66 | ♀ | 2017/03/05 | B | -2996bp/wt, KO positive | 28#/B6J | F1 |  |
| 68 | ♀ | 2017/03/05 | B | -2996bp/wt, KO positive | 28#/B6J | F1 |  |
| 69 | ♂ | 2017/03/09 | B | -3000bp/wt, KO positive | B6J/31# | F1 |  |
| 71 | ♀ | 2017/03/09 | B | -3000bp/wt, KO positive | B6J/31# | F1 |  |

ID, Identification number; ♀, female; ♂, male; B, Black; Gty, Genotype; DOB, Date of birth (year/month/date); Gen, Generation. F/M, father and mother.

**Table 3. Some F2, F3 generation of CCDC8-knockout mice**

| ID | Gender | Color | Gty | DOB | Gen |
| --- | --- | --- | --- | --- | --- |
| 149 | ♂ | B | -2996bp/wt | 2017/12/13 | N2F1 |
| 151 | ♂ | B | -2996bp/wt | 2017/12/13 | N2F1 |
| 153 | ♀ | B | -2996bp/wt | 2017/12/13 | N2F1 |
| 154 | ♀ | B | -2996bp/wt | 2017/12/13 | N2F1 |
| 155 | ♀ | B | -2996bp/wt | 2017/12/13 | N2F1 |
| 156 | ♂ | B | **-2996bp/-2996bp** | 2017/12/16 | N2F1 |
| 157 | ♂ | B | -2996bp/wt | 2017/12/16 | N2F1 |
| 159 | ♀ | B | -2996bp/wt | 2017/12/16 | N2F1 |
| 160 | ♀ | B | **-2996bp/-2996bp** | 2017/12/16 | N2F1 |
| 161 | ♀ | B | -2996bp/wt | 2017/12/16 | N2F1 |
| 173 | ♂ | B | -2996bp/wt | 2018/1/5 | N2F1 |
| 174 | ♀ | B | -2996bp/wt | 2018/1/5 | N2F1 |
| 195 | ♂ | B | -2996bp/wt | 2018/1/24 | N2F1 |
| 198 | ♀ | B | -2996bp/wt | 2018/1/24 | N2F1 |
| 200 | ♀ | B | -2996bp/wt | 2018/1/24 | N2F1 |
| 322 | ♀ | B | wt/wt | 2018/7/30 | N2F2 |
| 323 | ♂ | B | -2996bp/wt | 2018/8/1 | N2F2 |
| 324 | ♂ | B | -2996bp/wt | 2018/8/1 | N2F2 |
| 325 | ♂ | B | **-2996bp/-2996bp** | 2018/8/1 | N2F2 |
| 326 | ♂ | B | -2996bp/wt | 2018/8/1 | N2F2 |
| 327 | ♀ | B | -2996bp/wt | 2018/8/1 | N2F2 |
| 328 | ♀ | B | **-2996bp/-2996bp** | 2018/8/1 | N2F2 |
| 329 | ♂ | B | -2996bp/wt | 2018/8/6 | N2F3 |
| 330 | ♂ | B | wt/wt | 2018/8/6 | N2F3 |

Gty, Genotype; DOB, Date of birth (year/month/date); Gen, Generation.

♀, female; ♂, male; B, Balck.

**Table 4. Information of mouse breeding**

| Date | Generation | Total | Heterozygous CCDC8^+/-^ | Wild type | Homozygous CCDC8^-/-^ |
| --- | --- | --- | --- | --- | --- |
| 2018/01/05 | F2 | 104 | 57 | 45 | 2 |
| 2018/08/12 | F2/F3/F4 | 174 | 117 | 55 | 2 |
| 2020/01/09 | F5/F6/F7 | 132 | 105 | 27 | 0 |
| Total | | 410 | 279 | 127 | 4 |

**Table 5. Primers for PCR**

| Pair | Name | Sequence（5‘🡪3’） | GC% | Tm | Usage |
| --- | --- | --- | --- | --- | --- |
| 1 | Ccdc8-F1 | TCCCAGGCTTAGAGTGTAAG | 50.0 | 52.2 | Wt=3512bp |
|  | Ccdc8-R1 | CCACGGGCTTATGTCTTTCATTC | 47.8 | 62.8 | KO ~500bp |
| 2 | Ccdc8-F1 | TCCCAGGCTTAGAGTGTAAG | 50.0 | 52.2 | Wt=819 bp |
|  | Ccdc8-R2 | GGATGAGCAGATAGTCAACA | 45.0 | 50.1 | KO=none |
| 3 | Ccdc8-F3 | GCCTTTGTTAGCTACTGGGATG | 50.0 | 58.7 | Wt=646 bp |
|  | Ccdc8-R1 | CCACGGGCTTATGTCTTTCATTC | 47.8 | 62.8 | KO=none |
